# Synthesis and Characterization of Chemically and Green-Synthesized Silver Oxide Particles for Evaluation of Antiviral and Anticancer Activity

**DOI:** 10.3390/ph17070908

**Published:** 2024-07-08

**Authors:** Muhammad Asif, Wajeeha Iqbal, Muhammad Fakhar-e-Alam, Zahid Hussain, Malik Saadullah, Mudassir Hassan, Javed Rehman, Kholood A. Dahlous, Noora H. Al-Qahtani

**Affiliations:** 1 Department of Physics, Government College University Faisalabad, Faisalabad 38000, Pakistan; 2 Department of Pharmaceutical Chemistry, Government College University Faisalabad, Faisalabad 38000, Pakistan; 3 Department of Zoology, Government College University Faisalabad, Faisalabad 38000, Pakistan; 4 State Key Laboratory of Metastable Materials Science and Technology, School of Materials Science and Engineering, Yanshan University, Qinhuangdao 066004, China; 5 MEU Research Unit, Middle East University, Amman 11831, Jordan; 6 Department of Chemistry, College of Science, King Saud University, Riyadh 11451, Saudi Arabia; 7 Center for Advanced Materials, Qatar University, Doha P.O. Box 2713, Qatar

**Keywords:** silver oxide particles, *Datura innoxia*, anticancer activity, antiviral activity

## Abstract

Silver oxide (Ag_2_O) particles are wonderful candidates due to their unique properties, and their use in a wide range of research, industrial and biomedical applications is rapidly increasing. This makes it fundamental to develop simple, environmentally friendly methods with possible scaling. Herein, sodium borohydride and *Datura innoxia* leaf extract were applied as chemical and biological stabilizing and reducing agents to develop Ag_2_O particles. The primary aim was to evaluate the anticancer and antiviral activity of Ag_2_O particles prepared via two methods. XRD, UV-visible and SEM analyses were used to examine the crystallite structure, optical properties and morphology, respectively. The resulting green-synthesized Ag_2_O particles exhibited small size, spherically agglomerated shape, and high anticancer and antiviral activities compared to chemically synthesized Ag_2_O particles. The MTT (3-(4,5-dimethylthiazol-2-yl)-2,5 diphenyltetrazolium-bromide) assay of green-synthesized Ag_2_O particles showed high anticancer activity against MCF-7 cells with IC_50_ = 17.908 µg/mL compared to chemically synthesized Ag_2_O particles with IC_50_ = 23.856 µg/mL. The antiviral activity of green-synthesized Ag_2_O particles and chemically synthesized Ag_2_O particles was also evaluated by a plaque-forming assay, and green-synthesized Ag_2_O particles showed higher antiviral ability with IC_50_ = 0.618 µg/mL as compared to chemically synthesized Ag_2_O particles with IC_50_ = 6.129 µg/mL. We propose the use of green-synthesized Ag_2_O particles in cancer treatment and drug delivery.

## 1. Introduction

The global cancer problem is progressively increasing every year [1]. According to the World Health Organization (WHO) 2020 reports, there were 9.9 million deaths globally, with breast cancer mortality (2.26 million) being particularly high in developing countries, especially in Pakistan [2,3,4].

Metal oxide particles have been leading the way in materials research for industrial, energy, and biomedical applications over the last few decades.

A number of benefits, including ease of use, environmental friendliness, and ease of scaling, have generated considerable interest in green nanotechnology. Herbals are ingredients (extracts) that are derived from plants, either in combination or alone, and are used to treat and prevent many diseases. Many plant extracts have shown anticancer activity against several cancer cells without harming the growth of healthy cells. Plants are widely considered the optimal choice of material due to their stabilizing capabilities and bio-reducing properties [5,6].

Silver oxide particles have attracted significant interest among metal oxide particles, including gold, silver, manganese, zinc, and copper particles, because of their diverse range of uses in sectors such as textiles, cancer treatment, antiviral medications, electronics, food production, optical instruments, and rechargeable batteries and especially in biomedical applications [7,8,9,10]. Ag_2_O particles have antifungal [10], anticancer [11], antiviral [12], anticancer [13], anti-inflammatory [5], and antioxidant properties [14]. Ag_2_O particles can be prepared using a variety of techniques, including chemical, physical, and green-synthesis techniques [15,16,17]. Ag_2_O particles have high efficiency due to their outstanding antimicrobial activity towards viruses, bacteria, and other eukaryotic microorganisms [18,19]. Some important viruses are *influenza virus* [20], human *immunodeficiency virus (HIV)*, *human parainfluenza virus* [21], *dengue virus* [22], and *herpes simplex virus 1 (HSV1)* [23]. These viral infections are causing current epidemics and threaten the lives of humans on this planet [24,25].

In the current study, Ag_2_O particles were prepared via a green synthesis process by using *Datura innoxia* leaf extract. The extract effectively decreased and stabilized the ions present in the silver nitrate solution, resulting in Ag_2_O particle synthesis without requiring any chemical reducing or stabilizing agent. However, in the chemical synthesis process, sodium borohydride was used to prepare the Ag_2_O particles, which is a very strong stabilizing/reducing agent. The green and chemically prepared Ag_2_O particles were characterized. Finally, the anticancer and antiviral efficacy of green and chemically prepared Ag_2_O particles were explored. We suggest that further in vivo anticancer and antiviral studies of *Datura innoxia* Ag_2_O particles should be performed.

## 2. Results and Discussions

### 2.1. X-ray Diffraction Analysis

**Figure 1:**
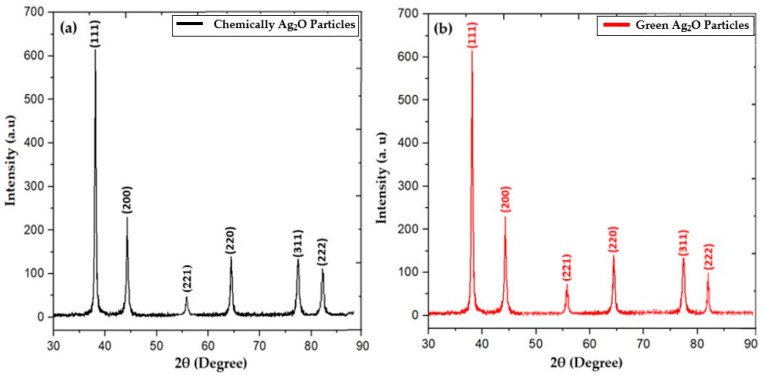
XRD graph of (**a**) chemically synthesized Ag_2_O particles and (**b**) green-synthesized Ag_2_O particles.

An X-ray diffraction (XRD) study was used to determine the crystal structure and calculate the crystallite size of Ag_2_O particles prepared using chemical and green synthesis processes. Figure 1a,b present the crystal structure patterns of the chemical and green synthesis of Ag_2_O particles prepared by coprecipitation processes and annealed at 400 °C for 12 h for refinement. The crystal structure of chemically and green-prepared Ag_2_O particles was investigated and calculated as cubic crystal structure and justified while comparing JCPDS No. 01-087-0719.The miller indices of maximum intensity peak are labeled with the (111) reflection/lattice plane at a diffraction angle of 38.4°, while the remaining lattice planes appeared in the following order: peaks at two thetas (2θ) = 44.5°, 64.6°, 77.6° and 82.3° labeled, and with respective indices of (200), (220), (311) and (222) in both chemical and green processes, respectively. The XRD pattern was similar in both conditions. In addition, it was observed that the maximum intensity peak had a broad size, which confirmed the growth of the particles. The average crystallite sizes of chemically and green-synthesized Ag_2_O particles were ~63 and ~54 nm, respectively, as shown in Table 1. Moreover, the crystallite size was calculated from X-ray diffraction (XRD) data using the given formulas [26,27,28,29].

(1) $$C.S=\frac{k\lambda }{\beta cos\theta }$$

Here, the crystallite size is (*C.S*), Scherrer constant (*k*), wavelength (*λ*), full width half maximum (*β*), and angle (*θ*).

### 2.2. FTIR Analysis

**Figure 2:**
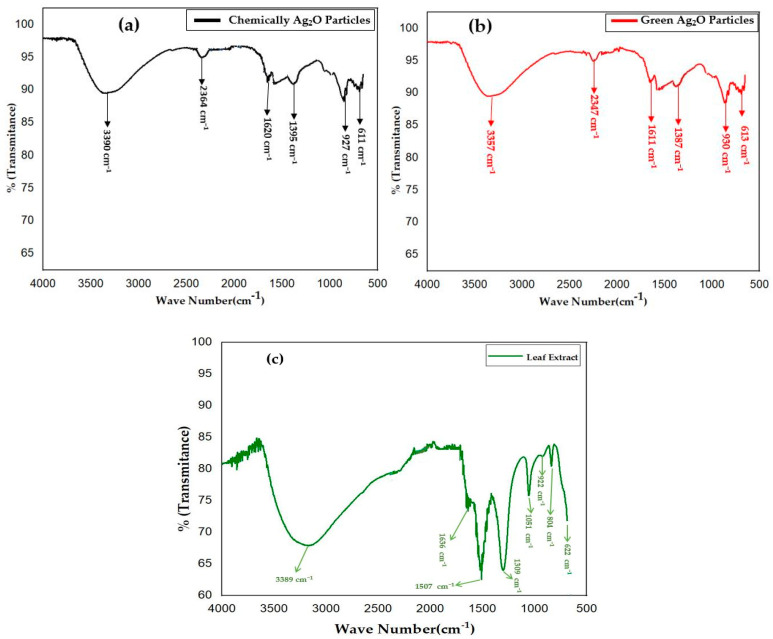
FTIR graph of (**a**) chemically synthesized Ag_2_O particles, (**b**) green-synthesized Ag_2_O particles, and (**c**) *Datura innoxia* leaf extract.

The chemical bond and functional groups of both chemically and green-synthesized Ag_2_O particles were investigated using Fourier transform infrared spectroscopy (FTIR) in the wavelength range of 500–4000 cm^−1^. In Figure 2a,b, it can be observed that the FTIR spectra of chemically synthesized Ag_2_O particles exhibited a shift in the bands compared to those synthesized using *Datura innoxia* plant extract. The addition of *Datura innoxia* plant extract to silver nitrate resulted in a change in the functional groups of Ag_2_O particles. The green-synthesized Ag_2_O particles showed several peaks corresponding to major functional groups at 3357, 2347, and 1631 cm^−1^, while the chemically synthesized Ag_2_O particles showed a shift in the bands at 3390, 2364, and 1620 cm^−1^. These peaks are associated with the stretching vibration of the hydroxyl group (H-bonded O-H stretch) [30,31,32]. Additionally, the O-H bending vibration of water molecules absorbed on the surface of Ag_2_O particles, which may be crucial for biomedical assays such as anticancer and antimicrobial activities [30,31], and a weak stretching vibration of C=C [17], were observed. The weak peaks at 1395 and 1387 cm^−1^ of Ag_2_O correspond to NO^3−^ ions (nitrates). Furthermore, the presence of an O-Ag-O bending mode of vibration at 611 cm^−1^ confirmed the formation of metal–oxygen bonding [9,30,31]. Moreover, the FTIR spectra of *Datura innoxia* leaf extract are shown in Figure 2c, which are quite similar to those in the previously published article [17].

### 2.3. UV–Visible Analysis

**Figure 3:**
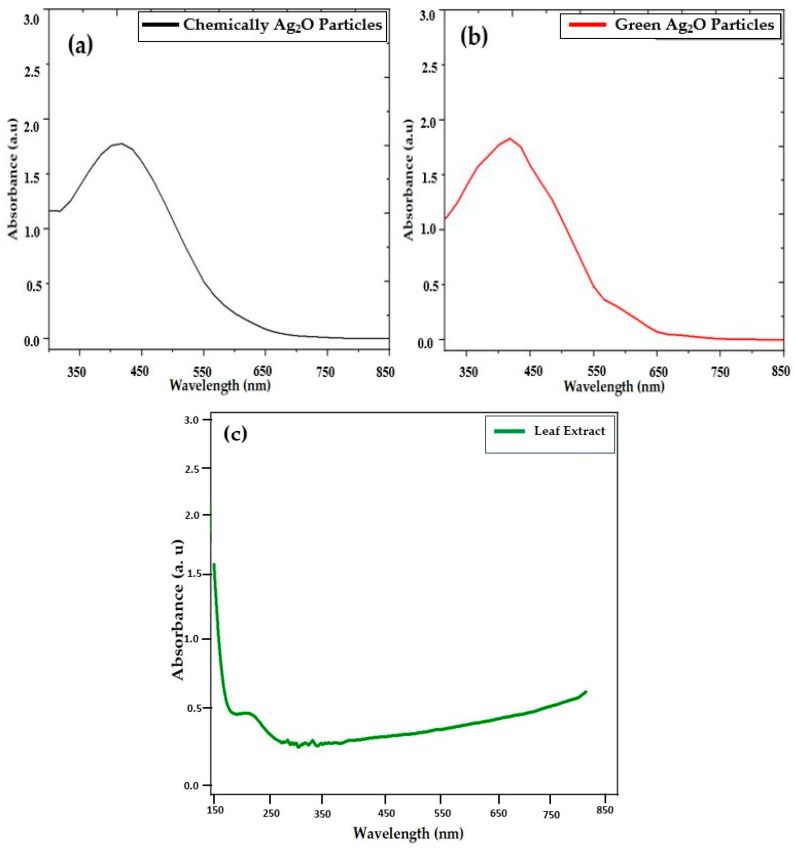
UV–visible graph of (**a**) chemically synthesized Ag_2_O particles, (**b**) green-synthesized Ag_2_O particles, and (**c**) *Datura innoxia* leaf extract.

UV–visible spectroscopy was employed to analyze the optical density or absorption spectra of both chemically and green-synthesized particles. In the current experimental strategy, chemically and green-synthesized Ag_2_O particles were carefully examined. When chemically and green-synthesized Ag_2_O particle samples were dispersed in deionized water to find the absorption spectra in the range of 350 nm to 850 nm, we revealed a sharp absorption band at 410 nm in the absorption spectra of chemically synthesized Ag_2_O particles, as depicted in Figure 3a. This might indicate the presence of chemically synthesized Ag_2_O particles such as in [17]. The absorption spectra of green-synthesized Ag_2_O particles are depicted in Figure 3b, where the presence of Ag_2_O particles is confirmed from the green-synthesized Ag_2_O sample using these spectra. Generally, green-synthesized Ag_2_O particles in deionized water absorbed radiation near the wavelength of 414.7 nm. It was observed from the UV–visible graph that the confirmation of the surface plasmon resonance peak near 425 nm indicates the presence of green-synthesized Ag_2_O particles, as shown in Figure 3b. On the metallic surface of green-synthesized Ag_2_O particles, there are resonant oscillations of free electrons through interbond transitions and surface plasmon resonance (SPR) bands [33]. In the graph, the surface plasmon peak indicates the presence of Ag_2_O particles with different sizes, and the spherical shape is confirmed in SEM. The absorption spectra of *Datura innoxia* leaf extract is depicted in Figure 3c, which shows a peak at ~220 nm. For more details about the absorbance of the *Datura innoxia* leaf extract, see the previously published paper [34].

### 2.4. SEM and EDX Analysis

**Figure 4:**
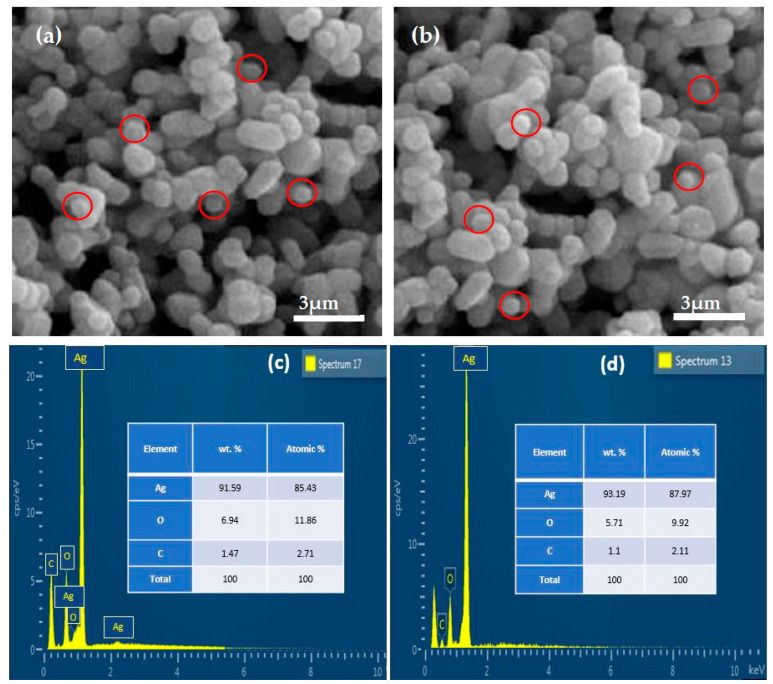
SEM images and EDX spectrum of (**a**) chemically synthesized Ag_2_O particles, (**b**) green-synthesized Ag_2_O particles at 3 µm, (**c**) chemically synthesized Ag_2_O particles, and (**d**) green-synthesized Ag_2_O particles.

The surface morphology of both chemically and green-synthesized Ag_2_O particles was analyzed using scanning electron microscopy (SEM). The SEM images of chemically and green-synthesized Ag_2_O particles are shown in Figure 4a,b at a scale of 3 µm scale, respectively. Spherical grains of the chemically and green-synthesized Ag_2_O particles can be seen in Figure 4, with some non-uniformly distributed aggregates present. The SEM micrographs confirmed that the chemically and green-synthesized Ag_2_O particles mostly have spherical shapes of various sizes. In Figure 4b, the bioagents of *Datura innoxia* leaf extract strongly interact with Ag ions to produce uniform spherical shapes with small sizes. The green-synthesized Ag_2_O particles had spherical shapes of dissimilar sizes but were smaller than the chemically synthesized Ag_2_O particles. The spherical grains were circulated with red circles. Hence, the SEM results of green-synthesized Ag_2_O particles were much better than those of the chemical Ag_2_O particles. The energy-dispersive X-ray spectroscopy (EDX) technique was employed to conduct an elemental compositional analysis of both chemically and green-synthesized Ag_2_O particles. The results from the EDX analysis verified the existence of Ag as a main element and O and C as the primary elements in the samples, as depicted in Figure 4c,d. The negligible percentage of carbon is due to lab environment or lab equipment. Kasthuri et al. [17] and Gajendaran et al. [35] also reported similar results, where elemental silver was detected in the graph obtained by EDX analysis, confirming the reduction of silver ions to elemental silver.

### 2.5. Anticancer Activity against MCF-7 Cells

**Figure 5:**
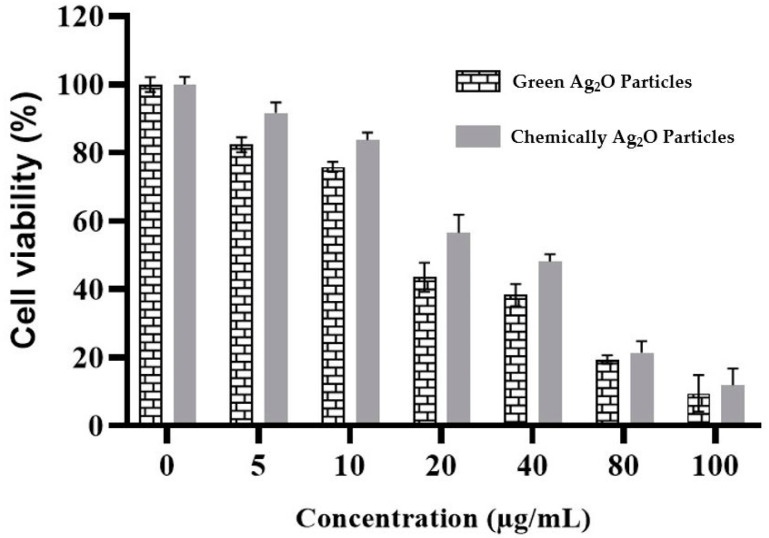
Cytotoxicity evaluation of both green- and chemically synthesized Ag_2_O particles against MCF-7 cell lines.

The results of the cytotoxicity investigation of both types of Ag_2_O particles on the breast cancer (MCF-7) cell line revealed that both particles were cytotoxic against the cancer cell line and were less toxic towards the Vero cell line. Maximum cell death was detected for the green-synthesized Ag_2_O particles with an IC_50_ of 17.908 µg/mL, compared to chemically synthesized Ag_2_O particles with an IC_50_ of 23.856 µg/mL against MCF-7 cells after 48 h of exposure. As can be seen in Figure 5, the cytotoxicity of both Ag_2_O particles is concentration-dependent [36].

### 2.6. Cytotoxicity Activity against Vero Cells

**Figure 6:**
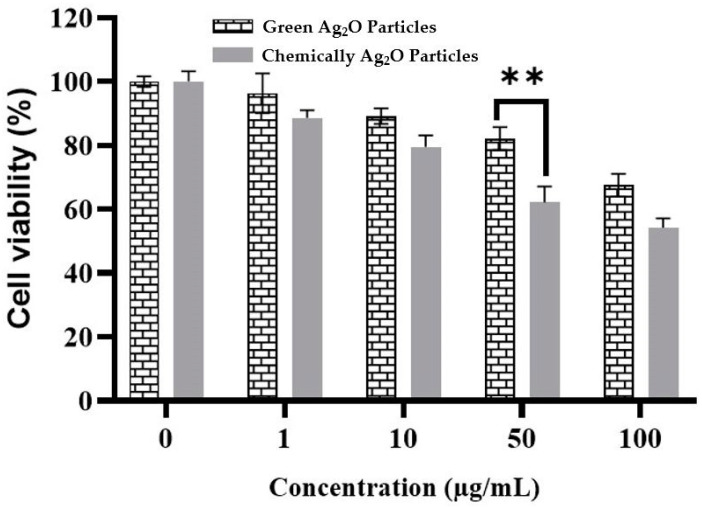
Cytotoxicity evaluation of both green and chemical Ag_2_O particles against normal (Vero) cell. ** *p* ≤ 0.01.

To rule out the possibility that cellular toxicity may be the cause of the decline in infectivity, a range of concentrations (1, 5, 10, 50, and 100 µg/mL) were incubated with Vero cell monolayers, as shown in Figure 6. Each concentration of Ag_2_O particles was incubated for various time periods (48 h), and cell viability was measured using the 3-(4,5-dimethyllthiazol-2-yl)-2,5 diphenyl-tetrazolium-bromide (MTT) assay. Chemically prepared Ag_2_O particles had toxic effects in the concentration range of 10–50 µg/mL. However, green-synthesized Ag_2_O particles within the range were shown/found to have low cytotoxicity in Vero cells. These statistics were valuable for determining concentrations for testing in antiviral assays. We used concentrations that were significantly below the cytotoxic threshold, ranging from 0.5 to 10 µg/mL.

### 2.7. Antiviral Activity

**Figure 7:**
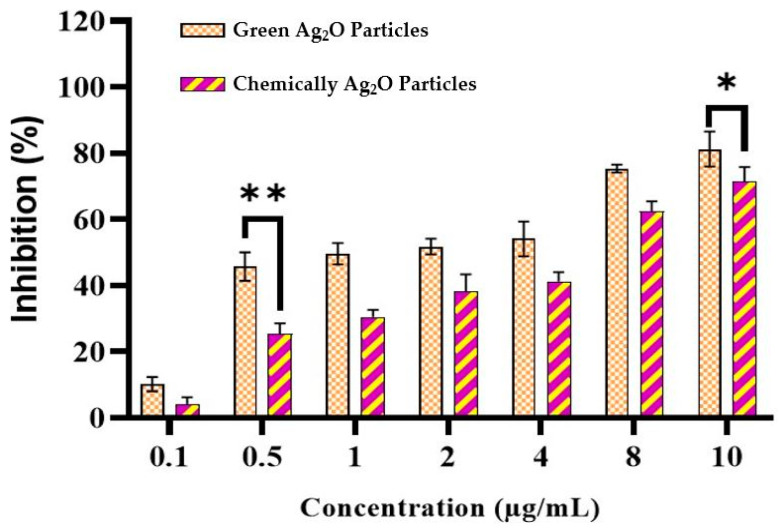
Green-synthesized Ag_2_O particles showed higher antiviral activity as compared to chemical-based Ag_2_O particles in a dose-dependent manner, where * is *p* ≤ 0.05, ** *p* ≤ 0.01.

A stock solution of silver oxide particles was prepared via a chemical-synthesis process as well as a green-synthesis process with a concentration solution of 10 mg/L by dispersing the working solution of silver oxide particles (0.1, 0.5, 1, 2, 4, 8 and 10 µg/mL) into Newcastle disease virus (NDV)-incorporated Vero cells. The solution was then removed, washed and inoculated with 0.5% methyl cellulose for 72 h. The medium was aspirated, and the cells were fixed with 10% formaldehyde and stained with 1% crystal violet; then, the plaques were counted. The plaque analysis formation was calculated. Green-synthesized Ag_2_O particles showed significant toxicity against NDV virus compared to chemically synthesized Ag_2_O particles (Figure 7).

### 2.8. Properties of Green-Synthesized Ag_2_O Particles

The Ag_2_O particles prepared using the green-synthesis process, which utilizes plant extracts, showed distinct properties compared to those prepared through traditional chemical methods. Some of the new properties of green-synthesized Ag_2_O particles are mentioned, such as smaller crystallite size, higher surface area, improved stability, increased antimicrobial and antioxidant potential, anticancer and antiviral properties, reduced toxicity and environmental impact, unique crystal structures and morphologies, potential for large-scale production, and cost-effectiveness. The green-synthesis method offers a sustainable and eco-friendly approach to producing Ag_2_O particles with advanced properties for various applications [6,12,13,16,17]. For more information on the properties of green-synthesized particles, see Table 2.

## 3. Materials and Methods

### 3.1. Materials

Silver nitrate (AgNO_3_), sodium borohydride (NaBH_4_), ethanol, polyvinylpyrrolidone, deionized water, dimethyl sulfoxide (DMSO), fetal bovine serum (FBS), Dulbecco’s modified eagle medium (DMEM), phosphate-buffered saline (PBS), and antibiotics (penicillin + streptomycin) were purchased from Sigma-Aldrich (St. Louis, MO, USA). In addition, the *Datura innoxia* plant was collected from the garden of the Department of Botany, Government College University, Faisalabad).

### 3.2. Preparation of Extract

Fresh leaves of *Datura innoxia* were cut into small pieces, washed twice with distilled water and dried [17]. In the next step, 200 g of powdered *Datura innoxia* was mixed with 750 mL of distilled water and heated at 80 °C for 3 h. Then, the obtained solution was filtered and scraped to obtain a dark yellow colored extract. Next, 0.407 g of extract was dissolved in 50 mL of deionized water and stirred continuously for 10 min for homogeneity.

### 3.3. Green Synthesis Method

**Figure 8:**
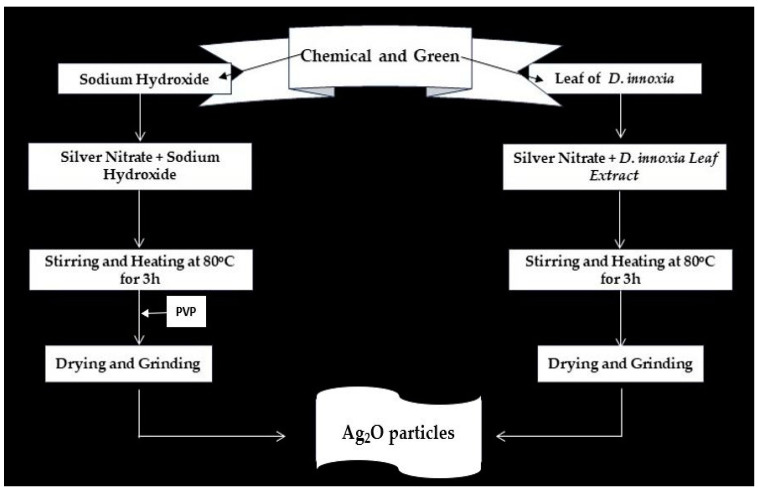
Schematic flow chart of chemically and green-synthesized Ag_2_O particles.

An amount of 3.5 g of AgNO_3_ was dissolved in 200 mL of distilled water. The prepared extract was added dropwise into the AgNO_3_ solution until the color of the solution turned dark brown and maintained its pH (9–11), and finally, we obtained the precipitates. The solution was centrifuged at 7000 rpm for 10 min and dried. Then, it was annealed at 400 °C for 12 h. Finally, the prepared precipitate was ground to obtain a fine powder [17,18,45]. A schematic flow chart is depicted in Figure 8.

### 3.4. Chemical Synthesis Method

An amount of 0.407 g of NaBH_4_ was dissolved in 50 mL of deionized water and kept in an ice bath for 3 h. In the next step, 3.5 g of AgNO_3_ was taken and dissolved in 200 mL of distilled water and kept under continuous stirring on a magnetic stirrer for 40 min to obtain a homogeneous solution [46]. Next, the NaBH_4_ solution was removed from the ice bath and placed in a magnetic stirrer, and then the AgNO_3_ solution was added dropwise. The solution was constantly stirred for 3 h to obtain dark-colored precipitates; then, 0.3 g Polyvinylpyrrolidone was mixed in 10 mL of water, and precipitates were prepared. The solution was centrifuged at 7000 rpm for 10 min and dried. Then, it was annealed at 400 °C for 12 h, and finally, the dark precipitate was ground to obtain a fine powder [27,47]. A schematic flow chart is depicted in Figure 8.

### 3.5. Characterization of the Synthesized Nanomaterial

X-ray diffraction (D8 advance Bruker, Karlsruhe, Germany) was used to confirm the crystal structure of the prepared particles. UV–visible spectroscopy (BK-UV1800PC DB, Bio-base, Jinan, China) was employed to compute the optical density, and FTIR (Spectrum 2, Perkin Elmer, Waltham, MA, USA) was used to study the functional groups (stretching and vibrational). The surface morphology of both chemically and green-synthesized Ag_2_O particles was analyzed using scanning electron microscopy (Cube 10 Emcraft, Hanam, Republic of Korea) analysis. An energy-dispersive X-ray (EDX) spectrometer was used to confirm the elemental composition of the synthesized materials.

### 3.6. Bioassay

#### 3.6.1. Cell Lines and Viruses

MCF-7 and green African monkey kidney (Vero) cell lines were gifted from the National Institute of Biotechnology and Genetic Engineering (NIBGE) in Faisalabad, Pakistan. MCF-7 and Vero cells were cultivated in DMEM/F-12 medium containing 1% (*v*/*v*) antibiotics and 10% (*v*/*v*) FBS. The flasks were kept at 37 °C in a humidified atmosphere with 5% CO_2_. NDV was grown in 9-day-old chicken embryo eggs. Before use, stock viruses were collected, titrated, and stored [46].

#### 3.6.2. Cytotoxicity Assay

MTT assays were used to measure the cytotoxicity of the chemically and green-synthesized Ag_2_O particles. MCF-7 cells were seeded in 96-well plates. Cells were incubated with increasing concentrations of particles at 37 °C and 5% CO_2_ for 48 h. The cells were treated with a 5 mg/mL MTT solution and then incubated for 4 h. After removing the MTT, 150 μL of DMSO was added and left for 5 min. The optical density was calculated using a microplate reader. Each experiment was performed in triplicate. The cytotoxicity level was quantified by determining the IC_50_ value, representing the concentration of particles that suppressed the growth of MCF-7 cells by half (50%) in comparison to untreated cells. Due to chemically synthesized Ag_2_O particles, the basicity of the solution may increase and hence become toxic towards the cell line. On the other hand, green-synthesized Ag_2_O particles were synthesized in a water-based extract, so they exhibited low toxicity. The percentage viability of Ag_2_O particles was calculated using the following formula [48,49].

(2) $$\%age\;Cell\;Viability=\frac{Abs\;of\;control-Abs\;of\;treated\;cells}{Abs\;of\;control}\times 100\%$$

#### 3.6.3. Antiviral Activity

Chemically and green-synthesized Ag_2_O particles were dissolved in DMEM without serum and used at concentrations of 0.5, 1, 2, 4, 8 and 10 µg/mL. The percentage (%) inhibition was calculated by dividing the number of plaques acquired in the positive control, which had 0% inhibition (no antiviral compounds added to the cell monolayers). A plaque-forming assay was performed to check the antiviral activity of both chemically and green-synthesized Ag_2_O particles. For this, confluent Vero cell monolayers (12-well plates) were cleaned with phosphate-buffered saline and infected with NDV for 1 h at 37 °C, with an infection diversity of 0.02 plaque-forming units per cell. The virus inoculum was mixed with Ag_2_O particles to perform the test. Unencapsulated virus was inactivated using citrate buffer at pH 3. After being washed in PBS, the samples were incubated for 48 h in fresh growing medium with carboxymethylcellulose. Monolayers infected with NDV were fixed and stained with 10% formaldehyde. The plates were then stained with crystal violet after fixation. Microscopically, the plaques were counted. The mean plaque count for each drug concentration was expressed as a percentage of the mean plaque count of the control virus, using the formula shown below. The plaques were quantified under a microscope. The mean plaque count for every drug concentration was calculated as a ratio to the mean plaque count of the control virus, using the formula below. A graph was plotted to show the relationship between the number of plaques and the drug concentration [29,49].

(3) $$Inhibition\;\left(\%\right)=\frac{\left(Mean\;no.\;of\;Plaques\;in\;control-Mean\;no.\;of\;Plaques\;in\;samples\right)}{\left(Mean\;no.\;of\;Plaques\;in\;control\right)}\times 100\%$$

### 3.7. Statistical Analysis (S.A)

Statistical analysis (S.A) was performed using Student’s *t*-test. All cytotoxic results on MCF-7 and Vero cell line experiments were reported as mean ± SD (standard deviation). A value of *p* ≤ 0.05 was statistically significant (indicated by *), and a value of *p* ≤ 0.01 was considered highly significant (indicated by **).

## 4. Conclusions

Silver oxide (Ag_2_O) particles were synthesized via a chemical- and green-synthesis approach. The XRD results revealed a cubic crystal structure. UV–visible spectroscopy results gave maximum absorption spectra at 410 nm and 414.7 nm, respectively, confirming the growth of chemically and green-synthesized Ag_2_O particles. An in vitro MTT assay of the green and chemically Ag_2_O particles revealed that both possess significant anticancer activity against MCF-7 cells with IC_50_ 17.908 µg/mL and IC_50_ 23.856 µg/mL, respectively. The antiviral activity of both particles was also evaluated by plaque-forming assay, and the green-synthesized Ag_2_O particles exhibited a higher antiviral ability with IC_50_ 0.618 µg/mL compared to chemically synthesized Ag_2_O particles with IC_50_ 6.129 µg/mL. These results revealed that green-synthesized Ag_2_O particles proved to be better than chemically synthesized Ag_2_O particles against cancer and viral infections. In the case of chemically synthesized Ag_2_O particles, toxicity (toxic behavior) was observed against normal (Vero) cells, unlike green-synthesized Ag_2_O particles. This observation further supports the idea that green-synthesized Ag_2_O particles are safer than chemically synthesized Ag_2_O particles. This research could potentially play a role in biomedical applications, particularly in the field of nanomedicine in the near future. We also suggest that in vivo anticancer and antiviral studies of green-synthesized Ag_2_O particles should be performed.

## Figures and Tables

**Table 1:** (**a**) shows the parameters, miller indices, diffraction angle, and crystallite size of chemically synthesized Ag_2_O particles. (**b**) shows the parameters, miller indices, diffraction angle, and crystallite size of the green-synthesized Ag_2_O particles.

Miller Indices (h,k,l)	Diffraction Angle 2θ (Theta)	Crystallite Size “*C.S*” (nm)
**(a)**		
(111)	38.1	41.3
(200)	44.3	55.7
(220)	64.5	75.6
(311)	77.4	81.2
**(b)**		
(111)	38.1	32.2
(200)	44.3	46.6
(220)	64.8	66.6
(311)	77.7	69.9

**Table 2:** Previous reported study of different plant extracts, nanoparticles, and their applications.

Sr#	Materials and Plants Name	Applications	IC_50_ (µg/mL)	References
1	Green-synthesized AgNPs (*Datura innoxia* leaf extract)	anticancer	15.2 and 8.5	[37]
2	Biosynthesis of AgNPs with *Datura innoxia*	anticancer	20	[28]
3	Green-synthesized AgNPs (*Baccaurea ramiflora*)	anticancer	110 and 140	[38]
4	AgNPs with *Syzygium aromaicum*	anticancer	60 and 50	[39]
5	Green-synthesized AgNPs (*Ctenolepis garcini*)	anticancer	33.78	[40]
6	Green-synthesized gold nanoparticles (AuNPs) with (leaf extracts of *Ocimum gratissimum Linn*)	anticancer	10 and 25	[41]
7	Green copper nanoparticles (CuNPs) with *Eclipta prostrata*	anticancer	1.71 and 1.81	[42]
8	PVP-coated AgNPs	antiviral	0.44	[43]
9	*Datura innoxia* leaf extract	anticancer	0.6	[44]
10	Green (*Datura innoxia* extract) and chemically synthesized Ag NPs	Anticancer, antibacterial and antioxidant	--	[17]

## Data Availability

Data is contained within the article.
